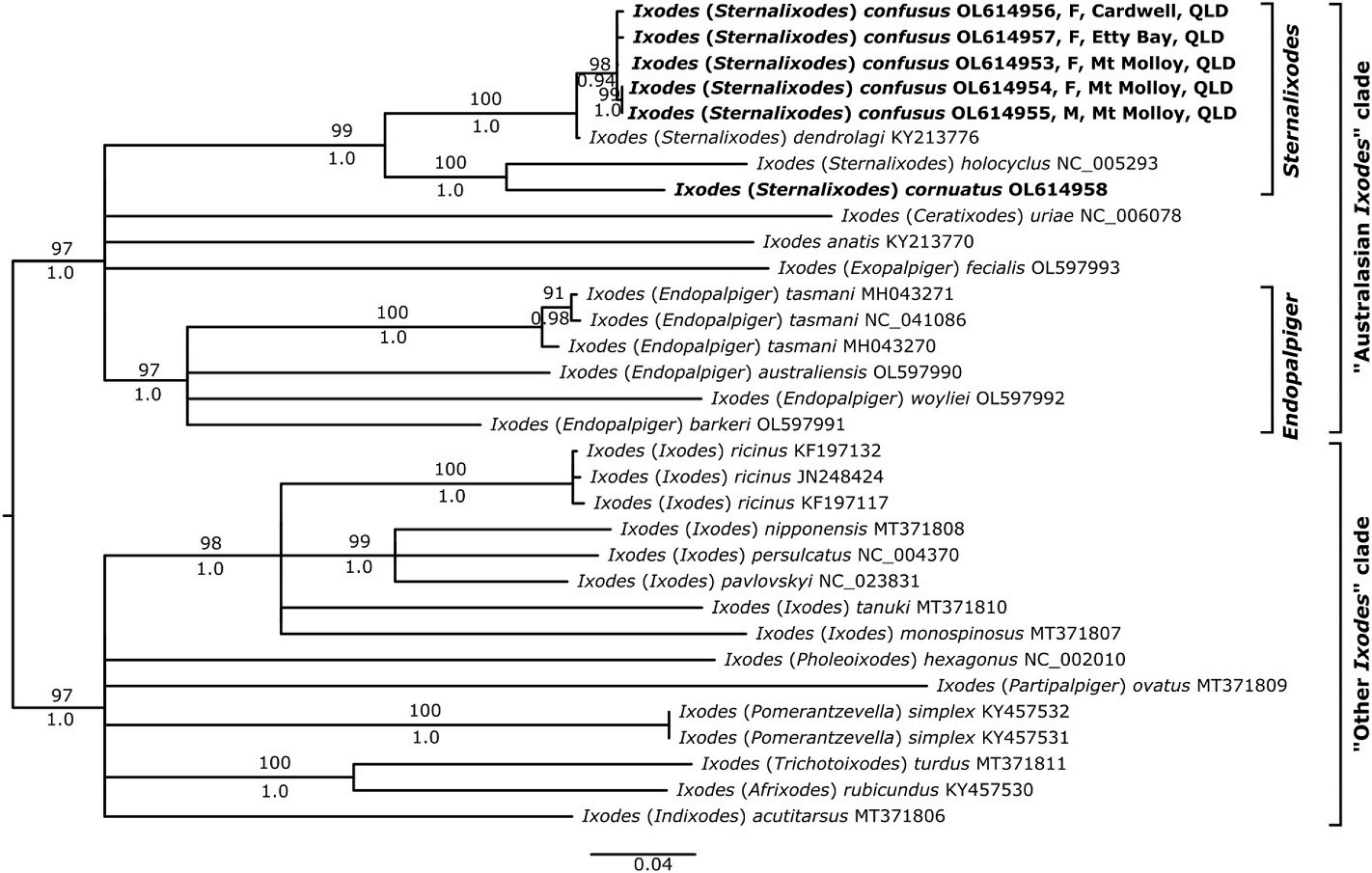# Rediscovery of *Ixodes confusus* in Australia with the first description of the male from Australia, a redescription of the female and the mitochondrial (mt) genomes of five species of *Ixodes*

**DOI:** 10.1016/j.ijppaw.2022.03.006

**Published:** 2022-03-22

**Authors:** Dayana Barker, Samuel Kelava, Owen D. Seeman, Renfu Shao, James R. Seaniger, Malcolm K. Jones, Maria A. Apanaskevich, Ryo Nakao, Dmitry A. Apanaskevich, Stephen C. Barker

**Affiliations:** a School of Veterinary Sciences, The University of Queensland, Gatton, Queensland, 4343, Australia; b Department of Parasitology, School of Chemistry and Molecular Biosciences, The University of Queensland, Brisbane, Queensland, 4072, Australia; c Queensland Museum, PO Box 3300, South Brisbane, 4101, Australia; d School of Science and Engineering, GeneCology Research Centre, University of the Sunshine Coast, Sippy Downs, Queensland, 4558, Australia; e Statesboro, GA, 30460, USA; f Laboratory of Parasitology, Department of Disease Control, Faculty of Veterinary Medicine, Hokkaido University, Sapporo, 060-0818, Japan; g US National Tick Collection, Institute for Coastal Sciences, Georgia Southern University, Statesboro, GA, 30460, USA; h Department of Biology, Georgia Southern University, Statesboro, GA, 30460, USA; i Zoological Institute, Russian Academy of Sciences, St. Petersburg, 199034, Russia

**Keywords:** Wet-tropics, Ixodida, Subgenus relationships, Tick, Systematics

## Abstract

We: (i) report the rediscovery of *Ixodes* (*Sternalixodes*) *confusus* Roberts, 1960 in Australia; (ii) redescribe the male and female of *I.confusus*; (iii) describe the mitochondrial (mt) genome of *I*. *confusus* from five ticks from four localities in Far North Queensland; and (iv) present the first substantial phylogeny of the subgenera of the *Ixodes*. The mt genomes of *I.confusus, I. cornuatus, I. hirsti, I. myrmecobii* and *I. trichosuri* are presented here for the first time*.* In our phylogeny from entire mt genomes (ca. 15 kb), the subgenus *Endopalpiger* was the sister-group to subgenera *Sternalixodes* plus *Ceratixodes* plus *Exopalpiger* whereas *Exopalpiger* was the sister to *Sternalixodes* plus *Ceratixodes*. [i.e. ((*Endopalpiger*) (*Sternalixodes, Ceratixodes* and *Exopalpiger*))]. Finally, we show that *Ixodes anatis*, the kiwi tick, may be closely related to the ticks of marsupials of Australia and Papua New Guinea*.*

## Highlights

• Rediscovery in Australia of the tick, *Ixodes confusus.*
• First description of the male of *Ixodes confusus* from Australia.
• Nine new mitochondrial genomes of *Ixodes* ticks from Australia and first substantial phylogeny of the subgenera of *Ixodes.*
• *Ixodes anatis*, a tick of the kiwi bird of NZ may be a closely related to the ticks of marsupials of Australia and PNG.

## Graphical abstract

**Figure:**
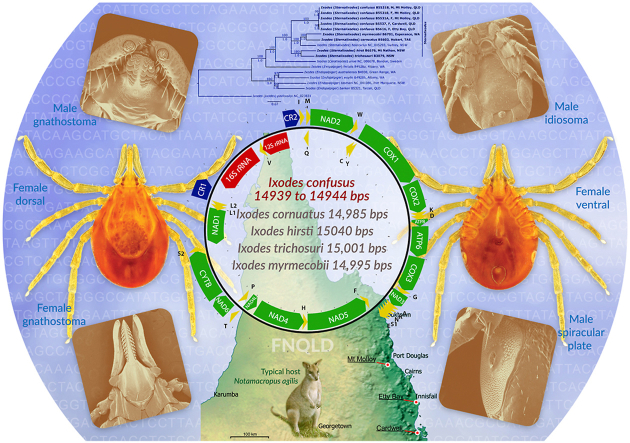

## 1 Introduction

The taxonomy of Australian ticks has received renewed interest during the past decade, invigorated by rigorous morphological and molecular techniques, and through public health concerns. Furthermore, part of this resurgence is a realisation that the work of Roberts (1970) was not the final word on Australian ticks and that much remained to be done. The focus of recent investigations has been the genus *Ixodes* Latreille, 1795, with new species described, either from novel collections (Ash et al., 2017; Kwak et al., 2018; Barker 2019) or from analyses of existing species (Heath and Palma 2017). However, good taxonomy also requires careful updating of previous descriptions, especially for taxa of uncertain taxonomic status. Among *Ixodes* of Australia, the species *Ixodes confusus* Roberts (1960) is a prime example.

*Ixodes confusus* was described from male and female ticks taken from wallabies in Sogeri, Papua New Guinea. Roberts (1955) initially considered the ticks to be *Ixodes cordifer* Neumann, 1908, a species with adults that typically parasitise cuscuses and possums in northern Australia, Papua New Guinea and Sulawesi (Roberts 1970; Wilson 1972). Later, Roberts (1960) realised that his specimens were a new species and, due to this confusion, named it *I. confusus.* The species is known from few specimens, yet Roberts (1970) considered the species to be common in Papua New Guinea on wallabies and cattle. However, in Australia *I. confusus* was known from just one female specimen taken from a human at Etty Bay near Innisfail, Queensland, in 1949. Thus, without further records in the following 70 years, the species may not be endemic, and the record could be from a human visitor from Papua New Guinea.

*Ixodes confusus* is one of eight species in the subgenus *Sternalixodes* Schulze, 1935 (Camicas et al., 1998). All eight of these species are endemic to the zoogeographic region Australasia. Five species occur only in Australia (*I*. *cornuatus* Roberts, 1960, *I*. *hirsti* Hassall, 1931; *I*. *holocyclus* Neumann, 1899; *I*. *myrmecobii* Roberts, 1962; *I*. *trichosuri* Roberts, 1960), one species only in Papua New Guinea (*I*. *dendrolagi* Wilson, 1967), and two species in both Australia and Papua New Guinea (*I*. *confusus*, *I*. *cordifer*). All of the species of *Sternalixodes* have a sternal plate in the female and nymph or only in the nymph (Roberts 1970; Wilson 1967). In addition, the females have a large scutum with strong lateral carinae; the palps are long and slender and the hypostome is lanceolate; the coxae are armed but lack syncoxae; and the anal grooves in the female meet at a point behind the anus. In the male, the anal plate is pointed posteriorly and the scutum has lateral grooves (Roberts 1970; Wilson 1967). Despite the morphological distinctiveness of the subgenus *Sternalixodes,* in general the subgeneric classification of *Ixodes* is controversial (Kwak and Heath, 2018; Barker et al., 2021) and requires further testing, particularly with genomic data. Nevertheless, some subgenera seem readily identifiable (Roberts 1960, 1970), and at least *Endopalpiger* Schulze, 1935 has both morphological and genomic support (Barker et al., 2022).

Herein, we show that *Ixodes confusus* is endemic to northern Queensland and provide a full description of the male and female*.* We also provide mitochondrial genomes for *I. confusus* and four other species of *Sternalixodes* (*I. cornuatus, I. hirsti, I. myrmecobii* and *I. trichosuri*) which further support the recognition of *Sternalixodes* as a distinctive subgenus of *Ixodes.*

## 2 Materials and methods

### 2.1 Material examined

Only field-collected ticks were available for study. The specimens were from new collections made by two of us (DB, SCB) from the Barker and Barker Collection (B&B) at the University of Queensland (UQ), the Queensland Museum (QM), the Australian National Insect Collection (ANIC) and the United States National Tick Collection (USNTC aka USNMENT, the US National Museum Entomology Collection) (Table 1).

**Table 1:** Specimens studied (morphology and/or genetics) in the present study. ANIC, Australian National Insect Collection; B&B, Barker & Barker Collection; QM, Queensland Museum; United States National Tick Collection (USNTC aka USNMENT, the US National Museum Entomology Collection). *, exact day of the month unknown.

Species	Host	Life stage/s	Place name	State	Date collected - be sure to have as 1953 and 2021	Latitude	Longitude	Altitude (m)	Postcode	Collection #	Collector	Collection
*I.* (*Sternalixodes*) *confusus*	“wallaby"	1M (holotype)	Sogeri (Mageri, Papua)	PNG	*.7.1953	−9.4211111	147.425215265454	500	na	G2456	J. Barrett	QM
*I. (Sternalixodes) confusus*	“wallaby"	1F (allotype, partly engordged)	Sogeri (Mageri, Papua)	PNG	from labels: 7.12.1953 & 7.7.1953	−9.41855936838451	147.4211111	500	na	G2455	J. Barrett	QM
*I.* (*Sternalixodes*) *confusus*	*Homo sapiens*	1F (paratype)	Etty Bay	Qld	24.3.1949	−17.589334	146.054309	6	4860	48 001 875	unknown	ANIC
*I.* (*Sternalixodes*) *confusus*	*Macropus agilis,* agile wallaby	2F	Etty Bay, patch of lowland rainforest in the vicininty of 195 Mourilyan Harbour Rd	Qld	*/11/2020	−17.589334	146.054309	6	4860	B5616	Zane Squarci, Tropical Veterinary Services, Innisfail	B&B
*I.* (*Sternalixodes*) *confusus*	vegetation (dry-ice trap)	1M	Etty Bay, patch of lowland rainforest in the vicinity of 195 Mourilyan Harbour Rd	Qld	15.1.2021	−17.589334	146.054309	6	4860	B5510	D Barker & S C Barker	B&B
*I.* (*Sternalixodes*) *confusus*	vegetation (dry-ice trap)	2F	near Etty Bay Caravan Park, Etty Bay	Qld	15.1.2021	−17.560393	146.089829	2	4858	B5511	D Barker & S C Barker	B&B
*I.* (*Sternalixodes*) *confusus*	*Thylogale stigmatica, red-legged pademelon*	1M 6F	Mt Molloy	Qld	23.1.2021	−16.627098	145.321381	410	4871	B5531b	Amber Mahlberg	B&B
*I.* (*Sternalixodes*) *confusus*	*Thylogale stigmatica*	1M 5F	Mt Molloy	Qld	23.1.2021	−16.627098	145.321381	410	4871	B6697	Amber Mahlberg	B&B
*I.* (*Sternalixodes*) *confusus*	*Macropus agilis, agile wallaby*	5F	Cardwell Forest, Bruce Highway, Damper Creek	Qld	28.1.2021	−18.380625	146.086528	725	4849	B5537	D Barker & S C Barker	B&B
*I.* (*Sternalixodes*) *confusus*	unknown	1F	Mossman River Gorge, Mossman	Qld	*.3.1948	−16.4737391233335	145.349488455844	61–152	4873	987518	Archibold Expedition	USNTC
*I.* (*Sternalixodes*) *confusus*	domestic cattle	3F	Sogeri (Mageri, Papua)	PNG	1970	−9.4211111	147.425215265454	500	na	987519	N.T. Talbot	USNTC
*I.* (*Sternalixodes*) *confusus*	domestic horse	1F	Sogeri (Mageri, Papua)	PNG	unknown	−9.4211111	147.425215265454	500	na	987495	N.T. Talbot	USNTC
*I.* (*Sternalixodes*) *confusus*	wallaby	3F	Sogeri (Mageri, Papua)	PNG	6.8.1957	−9.4211111	147.425215265454	500	na	987460	G.P. Holland	USNTC
*I.* (*Sternalixodes*) *confusus*	*Dorcopsis muelleri,* brown dorcopsis	1M	Vanapa River, Central Province	PNG	1970	−9.13333333	146.96666667	ca. 30	na	987471	N.T. Talbot	USNTC
*I.* (*Sternalixodes*) *cornuatus*	*Canis familiaris,* domestic dog	1F	Hobart	Tas	16.3.2021	−41.4545196	145.9706647	9	7008	B5603	Hobart community Veterinary hospital	B&B
*I.* (*Sternalixodes*) *mymrecobii*	*Canis familiaris,* domestic dog	1F	Esperance	WA	3.5.2021	−33.8502778	121.8830556	10	6450	B6701	Stephanie Hewlett, Swans Vet Services, Esperance, WA	B&B
*I.* (*Sternalixodes*) *hirsti*	*Felis catus*, domestic cat	1F	Winchelsea	Vic	1.12.2020	−38.243437	143.991368	ca. 100	3241	B6576	Geelong Animal Welfare Society, Vic	B&B
*I. (Sternalixodes) trichosuri*	*Trichosurus vulpecula,* common brushtail possum	1F	locality in NSW (precise locality unknown)	NSW	unknown	na	na	na	na	B3079	D.M. Spratt	B&B
*I.* (*Exopalpiger*) *fecialis*	*Isoodon obesulus,* southern brown bandicoot	1F	Albany	WA	20.10.15	−35.0518319219741	117.860731966204	ca. 10 m	6330	B4928a	Anne-Marie Horwitz & David Forshaw	B&B

### 2.2 Microscopy methods

Ticks were studied using a stereoscopic light-microscope (Nikon SMZ800N, Nikon Corporation, Tokyo, Japan and Olympus SZX16, Olympus Corporation, Tokyo, Japan) and a scanning electron microscope (TM4000 Plus Hitachi High-Technologies Corporation, Tokyo, Japan). Measurements are in millimetres and are given as the range followed by the mean and the number of specimens measured (n) in parentheses. Colour digital images were taken with a Canon 6D camera (Canon Corporation, Tokyo, Japan).

### 2.3 Sequencing and assembly of mitochondrial genomes

Tick DNA was extracted and prepared for sequencing at the University of the Sunshine Coast and UQ. Groups of ticks were cut in half and then incubated at 56 °C for 62 h with Proteinase K to lyse the cells. The QIAGEN DNeasy Blood and Tissue kit was used to extract genomic DNA. The amount of DNA recovered was measured with Nanodrop and Qbit instruments. Groups of ticks that yielded more than 200 ng of DNA were sent to Novogene Singapore for *de novo* library construction and next-generation Illumina sequencing. Groups of ticks with less than 200 ng were combined with DNA from a distinctly different organism, usually a bird, to reach the minimum threshold of 200 ng of DNA required by Novogene Singapore. At Novogene Singapore, DNA was sonicated to fragments, then fragments were end-polished, A-tailed and ligated with Illumina adaptors. DNA fragments were amplified with PCR, using P5 and P7 oligos, to create genomic libraries which were purified with AMPure XP system. The Illumina Novaseq 6000 sequencing platform was used to generate two giga-bases of nucleotide sequence data (PE 150). *De novo* contig assemblies of Illumina sequences were then constructed with Geneious Prime (Kearse et al., 2012). Blast-searches of contigs revealed mt genes of ticks; these gene sequences were then assembled until entire mt genomes had been assembled.

### 2.4 Annotation of mitochondrial genomes

Mitochondrial genomes were annotated with Geneious Prime. Protein-coding genes were identified by searches with BLAST (Chen et al., 2015) for open reading-frames. Regions between protein-coding genes were searched with BLAST (Chen et al., 2015) to find rRNA genes, tRNA genes and control regions. The tRNA that we expected to find but were not found with BLAST were found with the aid of the tRNAscan-SE Search Server v1.21 (Lowe and Chan 2016) and the MITOS Web Server (Bernt et al., 2013). The nucleotide sequences of tRNA genes were confirmed by studying the putative secondary structure of transcripts, as implemented in Geneious Prime (Kearse et al., 2012).

### 2.5 Phylogenetic methods

Phylogenies were inferred by both Maximum Likelihood (ML) and Bayesian Inference (BI) methods implemented in the RAXML-HPC2 v 8.2.12 (Stamatakis 2014) and MrBayes v3.2.2 (Ronquist et al., 2012) respectively. The sequence-alignment was put though Gblocks to remove regions with alignment gaps. JmodelTest2 v2.1.6 (Diego et al., 2012) was used to find the optimal substitution model for the nucleotide dataset. The GTR + G + I model was found to be the best fit for our dataset. In all ML and BI runs (experiments), genes were partitioned. Rapid-bootstrapping of 1000 replicates of our data was executed in RAXML-HPC2 v 8.2.12 (Stamatakis 2014). There were two simultaneous BI runs: 10 million generations sampled every 1000 MCMC steps. For every BI run, four MCMC chains (three heated and one cold) were executed. The first 25% of steps were discarded as burn-in. Tracer v 1.5 (Rambaut, 2009) was used to observe the effective sample size (ESS) and convergence of independent runs. Phylogenetic trees were displayed in FigTree v 1.4.4 (Rambaut, 2012). Branch support in the phylogenetic trees generated by RAXML-HPC2 v 8.2.12 (Stamatakis 2014) and MrBayes v 3.2.2 (Ronquist et al., 2012) was assessed by the bootstrap values and posterior probability values, respectively. All phylogenies were inferred through the CIPRES Science Gateway v.3.3 (Miller et al., 2010). *Ixodes* (*Ixodes*) *pavlovskyi* Pomerantzev, 1946, a species from the “other *Ixodes*” clade (*sensu* Barker et al., 2021) was the out-group.

## 3 Results

### 3.1 *Rediscovery of* Ixodes confusus *in Australia*

**Fig. 1:**
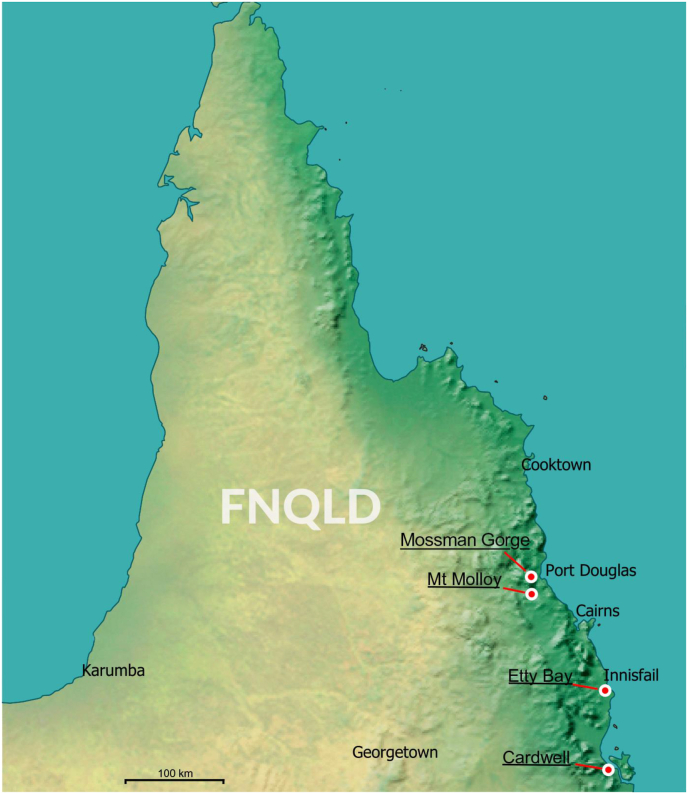
The five known localities in Queensland (Qld) Australia of *Ixodes confusus*Roberts (1960), indicated by white-with-red dots. Note that there were two sites at Etty Bay: (i) near Etty Bay Caravan Park; and (ii) Etty Bay, in the vicinity of 195 Mourilyan Harbour Rd, Etty Bay. (For interpretation of the references to colour in this figure legend, the reader is referred to the Web version of this article.)

*I.confusus* is now known at five sites in Far North Queensland: at two sites at Etty Bay near Innisfail, and at Mt Molloy, Cardwell and Mossman River Gorge, Qld (Table 1; Fig. 1).

### 3.2 Taxonomy and species redescription

Order **IXODIDA** Leach, 1815.

Superfamily **Ixodoidea** Banks, 1894.

Family **Ixodidae** Murray, 1877.

Genus ***Ixodes*** Latreille, 1795

Subgenus ***Sternalixodes*** Schulze, 1935.

#### 3.2.1 Redescription and diagnosis of Ixodes (Sternalixodes) confusus Roberts, 1960 (Fig. 2, Fig. 3, Fig. 4, Fig. 5)

*Ixodes confusus*Roberts, 1960, pp. 460–5, Figs 33 and 34.

**Fig. 2:**
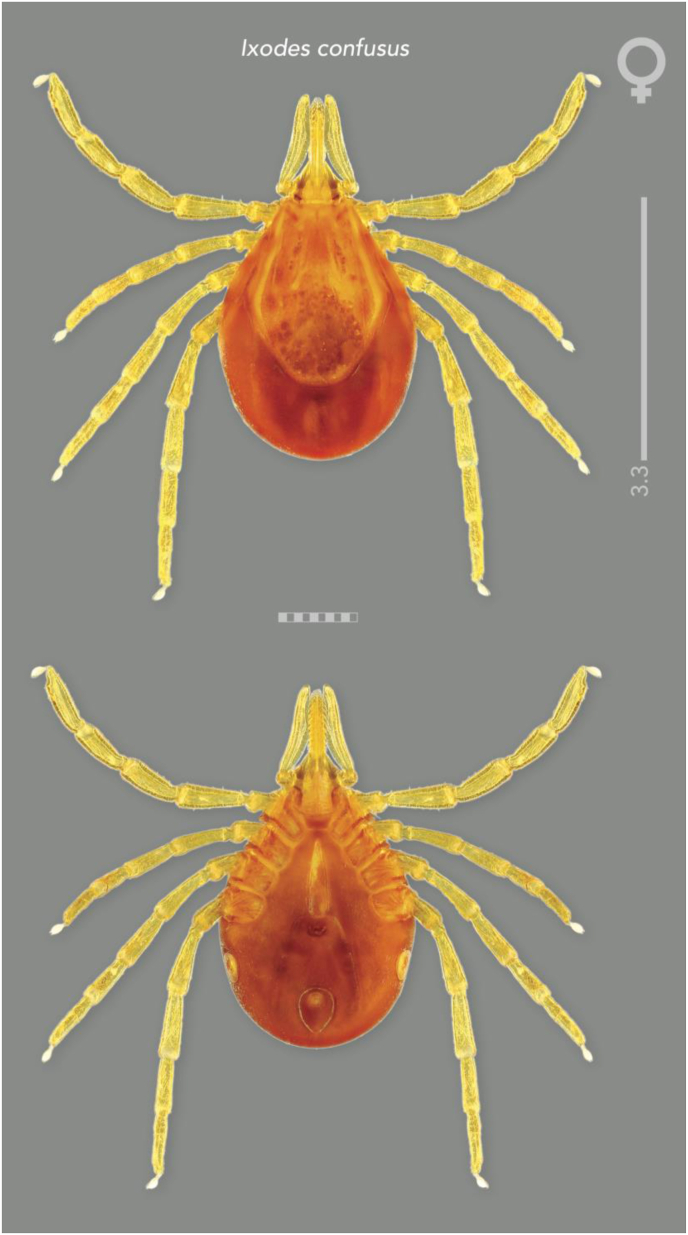
Paratype (female) of *Ixodes confusus*Roberts (1960) from Etty Bay, Queensland Australia (ANIC 48–001875), horizontal scale bar 1 mm, vertical scale bar 3.3 mm.

*Ixodes cordifer,*Roberts, 1955, pp. 21–5, Figs 1 and 2; 1959, p. 268.

#### 3.2.2 Type-host

“wallaby” (Diprotodontia: Macropodidae).

#### 3.2.3 Other hosts

*Dorcopsis muelleri* (Lesson), *Macropus agilis* (Gould), *Thylogale stigmatica* (Gould) (Diprotodontia: Macropodidae); *Equus caballus* Linnaeus (Perissodactyla: Equidae); *Bos taurus* Linnaeus (Artiodactyla: Bovidae).

#### 3.2.4 Type-locality

Sogeri, Central Province, Papua New Guinea.

#### 3.2.5 Type-material

Holotype male, Sogeri (∼9°25′S, 147°26′E), Central Province, Papua New Guinea, July 1953, in QM (G2456). Host: “wallaby".

Allotype. Female (partly engorged), Sogeri, Papua New Guinea, 7. xii.1951 (J. Barrett), in QM (G2455). Host: “wallaby".

Paratypes. Roberts (1960 p. 460) wrote that the following paratypes were in the Animal Research Institute, which now known as the Qld Government Department of Agriculture and Fisheries, Brisbane, and the Veterinary Parasitology Laboratory, C.S.I.R.O.,

Yeerongpilly, Qld (this collection is now part of the ANIC) but we did not find these paratypes in these places so they are at present, lost: 1M, 3F (partly engorged), Sogeri, Papua New Guinea, 28. i.1951 (J. Barrett), “wallaby”; 1F (partly engorged), 1F (fully engorged), same data as allotype; 2F (partly engorged), same data as holotype; 3F (partly engorged), Sogeri, Papua New Guinea, 10. xi.1951 (J. Barrett), “wallaby”. We did, however, find a paratype from Etty Bay, Far North Queensland in the ANIC (48 001 875): 1F (unfed), Etty Bay, Far North Queensland, Australia, 24. iii.1949, man.

Other material (collected by us). Twenty-four adults (3M, 21F). Australia, Far North Queensland: Etty Bay, Mourilyan Harbour Rd; Etty Bay Caravan Park, Etty Bay; Mt Molloy; Cardwell Forest, Bruce Highway, Damper Creek (see Table 1 for details).

#### 3.2.6 Etymology

In the words of Roberts (1970 p. 53) “*I. confusus* was confused with *cordifer* by Roberts (1955); hence the name *Ixodes confusus* Roberts, 1960”.

#### 3.2.7 Redescription

**Male** [based on the 3 most complete specimens: Etty Bay (B5511), Mt Molloy (B5522, B6697), Qld, Australia] Idiosoma (Fig. 4A and B) broadly elongate-oval, widest slightly posterior to mid-length; length from apices of scapulae to posterior body margin 2.41–2.65 (2.56), width 1.68–1.90 (1.76), ratio 1.39–1.55 (1.46). Lateral groove distinct. Conscutum (Fig. 4A) length 2.31–2.64 (2.48), width 1.62–1.66 (1.63), ratio 1.43–1.59 (1.52); scapulae short, blunt; lateral carinae as rounded ridges; cervical grooves shallow; punctations fine, shallow, moderately dense in central field, punctations larger, deeper and denser on lateral fields and pseudoscutum; setae sparse, short (0.01) and distinct. Ventral plate outlines as illustrated (Fig. 4B); median plate: length 1.20–1.50 (1.32), width 0.98–1.25 (1.07), ratio 1.20–1.28 (1.24); adanal plate: length 0.53–0.68 (0.58), width 0.48–0.67 (0.57), ratio 0.95–1.16 (1.04); anal plate: length 0.48–0.50 (0.48), width 0.48–0.50 (0.50), ratio 1.00–1.00 (1.00). All ventral plates with fine to small, sparse punctations (Fig. 4). Genital aperture (Fig. 4B) located at level of posterior margin of coxae II; posterior margin of genital apron deeply concaved. Ventral setae (Fig. 4) sparse, short, evenly distributed on all plates; length of setae on median plate (0.03). Anal groove (Fig. 4C) slightly convex anteriorly and closed posteriorly. Spiracular plate (Fig. 4D) broadly oval, longer than wide, length 0.40–0.67 (0.54), width 0.15–0.38 (0.27), ratio 1.79–2.67 (2.15).

**Fig. 3:**
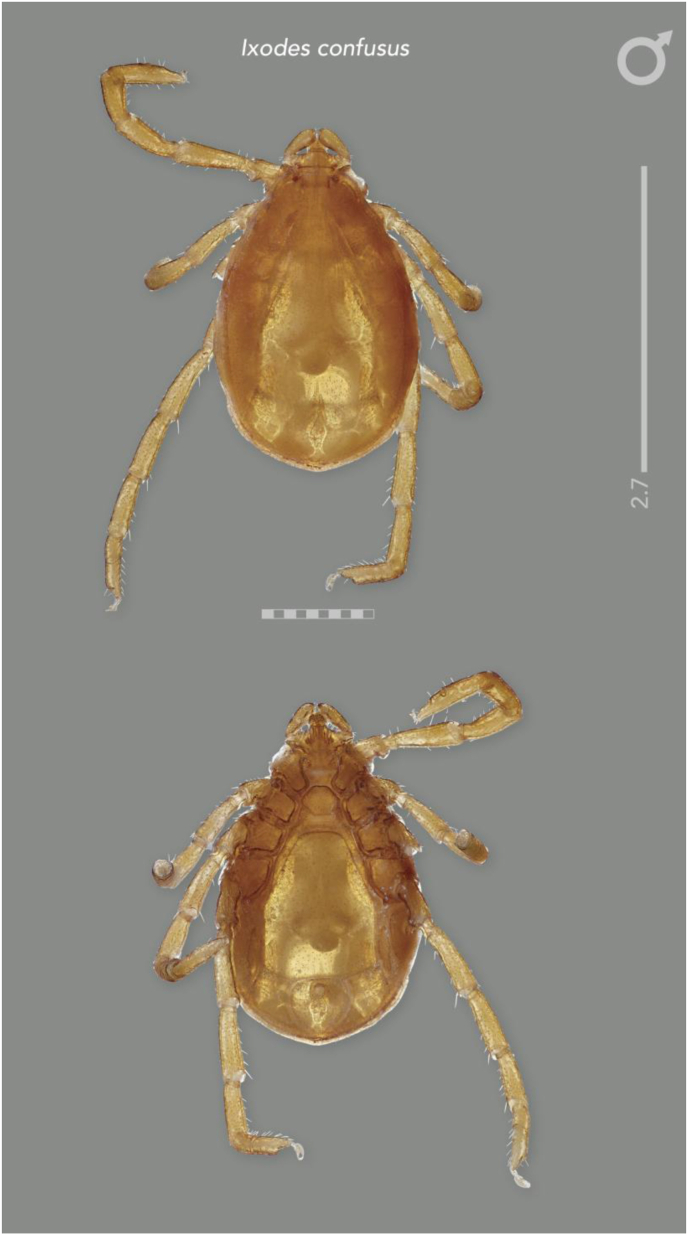
Holotype (male) of *Ixodes confusus*Roberts (1960) from Sogeri, Papua New Guinea (QM QM-G2456), horizontal scale bar 1 mm, vertical scale bar 2.7 mm.

**Fig. 4:**
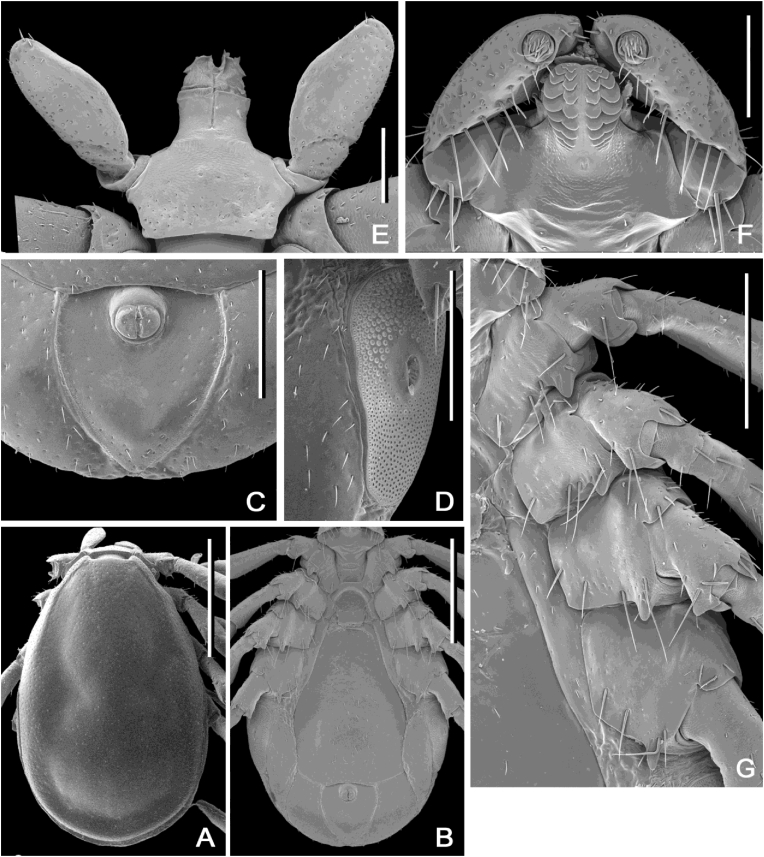
*Ixodes confusus*Roberts (1960), scanning electron micrographs of **male**. A, Idiosoma, dorsal view; B, Idiosoma, ventral view; C, Anal plate; D, Spiracular plate; E, Gnathosoma, dorsal view; F, Gnathosoma, anteroventral view; G, Coxae. *Specimens*: A, B6697 Mt Molloy, Qld; B, C, D, F, G B5511 Etty Bay; E, B5537 Cardwell. *Scale-bars*: A, B, 1 mm; C, D, 0.4 mm; E, F, 0.2 mm; G, 0.5 mm.

Gnathosoma (Fig. 4E and F) length from palpal apices to posterior margin of basis capituli dorsally 0.53–0.60 (0.56, width between lateral projection of palpal segments I 0.45–0.63 (0.55), ratio 0.91–1.22 (1.03). Dorsal basis capituli (Fig. 4E) subrectangular, with converging lateral margins; cornua indistinct. Ventral basis capituli (Fig. 4B) subrectangular; lateral margins with slight constrictions at mid-length; auriculae absent; pair of transversal ridges in auriculae area. Palpi (Fig. 4E and F) short, length dorsally (segments II-III) 0.38–0.45 (0.41), maximum width (in dorsolateral plane) 0.15–0.23 (0.19), ratio 1.83–2.50 (2.19); segment I subtriangular ventrally; suture between segments II and III indistinct, narrower proximally, gradually widening to mid-length and then gradually converging to broadly rounded apex. Hypostome (Fig. 4F) length 0.23–0.30 (0.27), width 0.18–0.23 (0.20), ratio 1.13–1.71 (1.35); club-shaped, widening to broadly rounded apex; base of hypostome at level of base of palpal segment II; dental formula 2/2, denticles rounded.

Legs moderately long, slender. Coxae (Fig. 4G): coxae I-IV with moderately long and narrow external spur with narrowly rounded apex; spur on coxae I-III subequal, spur on coxa IV slightly longer than those on coxae I-III; coxae I-IV without syncoxae. Trochanters I-II with indistinct spur ventrally; trochanters III-IV with short, narrow, narrowly rounded at apex spur ventrally. Tarsus I: length 0.60–0.83 (0.72); tarsus IV length 0.58–0.70 (0.66); tarsi humped subapically.

**Fig. 5:**
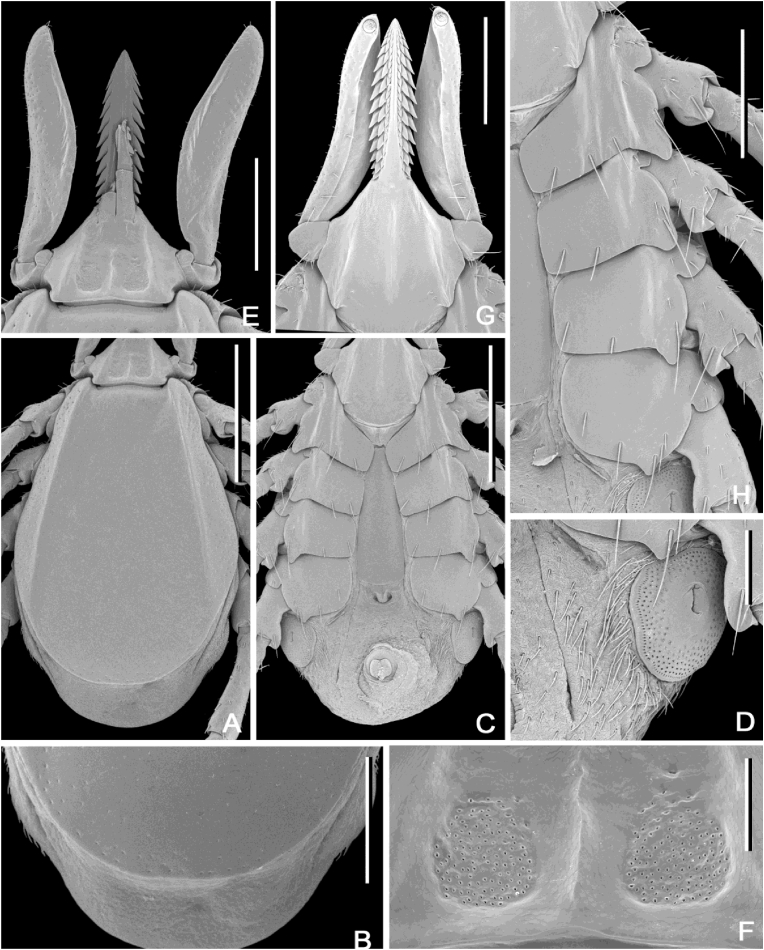
*Ixodes confusus*Roberts (1960), scanning electron micrographs of **female**. A, Idiosoma, dorsal view; B, Idiosoma showing posterior portion of scutum and alloscutum; C, Idiosoma, ventral view; D, Spiracular plate; E, Gnathosoma, dorsal view; F, Porose areas; G, Gnathosoma, ventral view; H, Coxae. *Specimens*: A-H, B5510 Etty Bay, Qld. *Scale-bars*: A, C, 1 mm; B, E, G, H, 0.5 mm; D, F, 0.2 mm.

**Female** [based on the 3 most complete specimens: Etty Bay (B5510, 2 individuals), Cardwell (B5537), Qld, Australia]. Idiosoma (Fig. 5) length from scapular apices to posterior body margin in slightly engorged specimens 2.55–2.94 (2.70), width in moderately engorged specimens 1.65–2.23 (1.98), ratio 1.15–1.59 (1.38.), broadly oval, widest approximately at mid-length in moderately engorged specimens. Scutum (Fig. 5A) length 2.28–2.48 (2.35), width 1.65–2.05 (1.87), ratio 1.21–1.38 (1.26); lateral margins diverging for slightly more than 1/2 of scutum length, then converging to broadly rounded margin posteriorly; scapulae blunt; lateral carinae well-developed, as sharp ridges reaching posterior margin of scutum; cervical grooves shallow; punctations fine and sparse in central field, small and dense on lateral fields and along posterior margin of scutum; setae short, indistinct. Alloscutum as illustrated (Fig. 5A); setae of alloscutum short 0.02–0.04 (0.03) and evenly distributed. Venter (Fig. 5C) as illustrated; genital aperture (Fig. 5C) medial to coxae IV; sternal plate long and subtriangular (Fig. 5C); anal groove (Fig. 5C) joined posteriorly forming pointed apex; ventral setae moderately long and dense, longer and denser around spiracular plates. Spiracular plates (Fig. 5D) length 0.38–0.65 (0.51), width 0.27–0.55 (0.46), ratio 0.88–1.25 (1.10); angularly suboval.

Gnathosoma (Fig. 5E and G) length from palpal apices to cornual apices, dorsally 1.20–1.30 (1.25), width between lateral projection of palpal segments I 0.95–1.03 (0.98), ratio 1.23–1.32 (1.27). Dorsal basis capituli (Fig. 5E) subrectangular, posterior margin nearly straight; cornua very short, triangular, with blunt apex; pair of sharp ridges extending from anterolateral margins of basis capituli to cornual apices; short ridge between porose areas; porose areas distinct, with clearly circumscribed borders, subcircular, separated by distance slightly less of their own width. Ventral basis capituli (Fig. 5G) pentagonal; auriculae short, triangular with narrowly rounded apex; pair of sharp ridges extending from anterolateral margins of basis capituli to auriculae apices; sharp median ridge extending from base of hypostome to level of auriculae on basis capituli. Palps (Fig. 5E and G) slender, length dorsally (segments II-III) 0.98–1.15 (1.08), maximum width (in dorsolateral plane) 0.20–0.23 (0.22), ratio of 4.33–5.50 (4.98); segment I subtriangular with broadly rounded corners ventrally; suture between segment II and III indistinct; segment II and III narrower proximally, gradually widening to midlength and then gradually converging to broad apex.

Hypostome (Fig. 5E and G) length 0.75–0.83 (0.78), width 0.20–0.25 (0.23), ratio 3.30–3.88 (3.50); lanceolate, sharply pointed; dental formula appears mostly 3/3 (some rows may be 4/4): inner most files have more denticles (approximately 16–21) than median and lateral files.

Legs moderately long, slender. Coxae (Fig. 5H): coxae I–IV with moderately long, broadly triangular external spur with blunt apex; spur on coxa I slightly longer than those on other coxae, spur on coxae II and III subequal, spur on coxa IV nearly twice shorter than on other coxae; coxae I–IV with distinct longitudinal ridges laterally. Trochanters I-IV with distinct but small triangular spur ventrally (not obvious on SEMs but can be seen by light microscopy). Tarsus I: length 0.80–1.03 (0.94 n = 3); tarsus IV length 0.75–0.88 (0.83); tarsi slightly humped subapically.

### 3.3 Remarks and differential diagnosis

Roberts (1955, 1970) had much trouble distinguishing *I. confusus* and *I. confusus*, particularly the males. Since both *I. confusus* and *I. confusus* are now known to be present in Far North Queensland there was the possibility that we might inadvertently associate a male of *I. confusus* (rather than a male of *I. confusus*) with the females of *I. confusus*. Therefore, we sequenced the mt genome of a putative male of *I. confusus* from Mt Molloy so that the sequence of this male could be compared with the sequence of the females we sequenced from Mt Molloy, Etty Bay and Cardwell. The male sequence matched the females well (99.6%) confirming that the collected males were indeed *I. confusus*. Alas, our attempts to sequence the mitochondrial genome of *I.* *cordifer* from Far North Queensland and Papua New Guinea failed. However, our morphological study supported the separation of *I. confusus* from *I. confusus*.

In the male of *I. confusus* the spur on coxa IV is only a little longer than that on coxa III, and the spurs on trochanters III and IV are stout, whereas in *I. confusus* the spur on coxa IV is much longer than spur on coxa III, and the spurs on trochanters III and IV are slender. The female of *I. confusus* can be easily distinguished from that of *I. cordifer* by the former having ridges on the dorsal (one ridge) and ventral (three ridges) basis capituli and ridges on coxae I-IV. Furthermore, in *I. confusus* the sternal plate is almost triangular (Fig. 5C) whereas in *I. cordifer* it is almost rectangular; the males of these species are also distinguished in the keys of Barker (2019).

### *3.4* *Mitochondrial* (*mt*) *genomes of* Ixodes confusus *and its relatives*

**Fig. 6:**
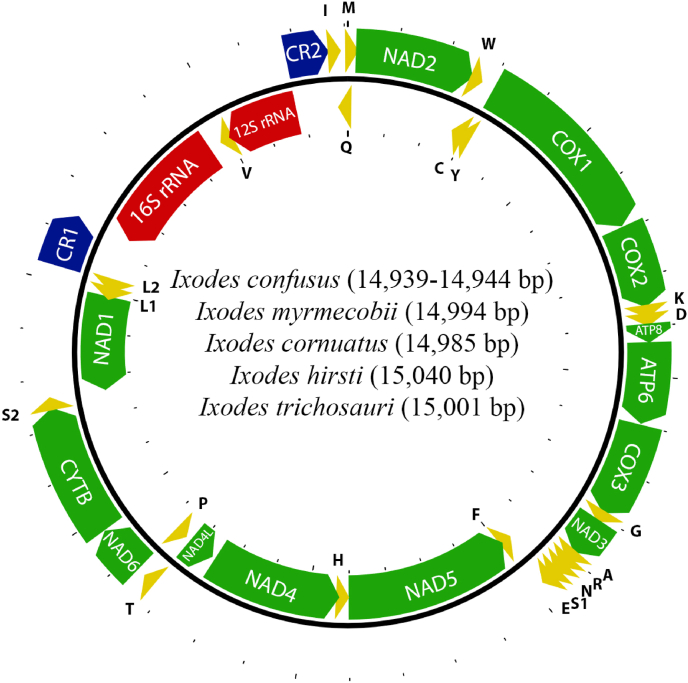
The mitochondrial genomes of *Ixodes* (*Sternalixodes*) *confusus*, *I.* (*St.*) *myrmecobii*, *I.* (*St.*) *cornuatus, I.* (*St.*) *hirsti* and *I.* (*St.*) *trichosuri.* Protein-coding genes are shown in green, tRNAs are in yellow, rRNAs are in red whereas the two control regions are in blue. Protein-coding genes are labelled by their four-character abbreviations, tRNAs are labelled by their one-letter amino acid abbreviations whereas the two control regions are labelled as CR1 and CR2. Variation in the size of mitochondrial genome is indicated in parenthesis. (For interpretation of the references to colour in this figure legend, the reader is referred to the Web version of this article.)

**Fig. 7:**
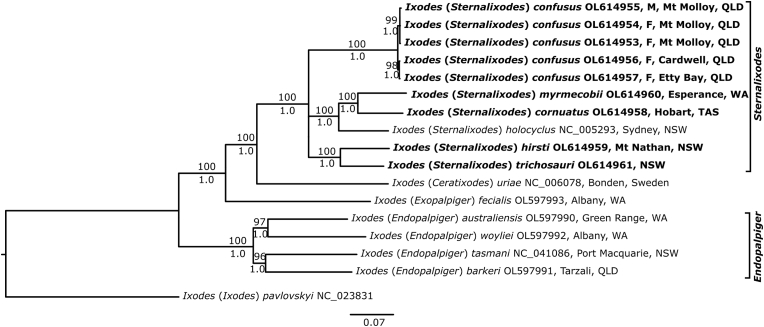
Maximum likelihood (ML) phylogenetic tree from entire mt genomes (15,254 bp alignment). Tip-labels indicate NCBI accession numbers. Numbers above branches show Maximum Likelihood bootstrap support whereas numbers below branches show the Bayesian Posterior Probability support. *Ixodes pavlovskyi* Pomerantzev, 1946, one of the species of “other *Ixodes*” clade (*sensu*Barker et al., 2021), for which an entire mitochondrial (mt) genome was available in GenBank, was set as the outgroup. The scale bar indicates 0.07 nucleotide substitutions per nucleotide site for the 15,254 nucleotide sites in our alignment of theses entire mt genomes. So, for example, there were about 1067 nucleotide substitutions along the branch that leads to *I.* (*Ceratixodes*) *uriae* plus *I.* (*Sternalixodes*) *holocyclus* plus *I.* (*Exopalpiger)fecialis*, which is marked with an asterisk (i.e. 0.07 nucleotide substitutions per nucleotide site x 15,254 nucleotide sites (bps) = 1067 nucleotide substitutions). Ticks in bold were sequenced in the present study.

The mt genomes of five species are presented here for the first time: *I.* (*Sternalixodes*) *confusus*, *I.* (*St.*) *cornuatus*, *I.* (*St.*) *hirsti*, *I.* (*St.*) *trichosuri* and *I.* (*St.*) *myrmecobii* (Fig. 6). The mt genomes of *I. confusus*, *I. cornuatus*, *I. hirsti, I. trichosuri* and *I. myrmecobii* have the gene-arrangement that is typical of *Ixodes* that are endemic to Australia (Barker et al., 2021, Fig. 1). A phylogeny from these mt genomes, together with previously published mt genomes of *Ixodes*, indicate monophyly of the subgenera *Sternalixodes* and *Endopalpiger* (Fig. 7). The mitochondrial genomes published for the first time in this paper have been submitted to GenBank database; accession numbers OL614953 to OL614959.

## 4 Discussion

### *4.1* *Rediscovery of* Ixodes (Sternalixodes) confusus *in Australia*

Before our study, *I. confusus* was known in Australia from a single specimen collected from a human at Etty Bay in 1949 (Table 1; Roberts 1960, 1970). Thus, it was extremely doubtful that *I.* c*onfusus* was endemic to Australia. Indeed, the single female specimen could have been brought to Australia from Papua New Guinea on a human, since humans often travel between Papua New Guinea and Cairns, and not a single adult *I. confusus* had since been collected in Australia. However, a concerted collection effort (DB, SCB) demonstrated that this species is present in Far North Queensland, where it is probably widespread (Table 1; Fig. 1). The host records suggest adults have a preference for macropods (27/35 ticks) but the records from a human, horse and cattle show *I. confusus* occasionally attaches to other hosts. [We recently found another female in the USNTC from Mossman River Gorge, Mossman, Qld, which was collected during an Archibold Expedition in 1948 (Table 1).]

### *4.2* *Mitochondrial (mt*) *genomes of* Ixodes confusus*, four of its relatives, and the phylogenetic position of the subgenus* Sternalixodes

The live *I. confusus* provided the first opportunity to investigate the phylogenetic relationships of *I. confusus* to its relatives with large numbers of nucleotides. Previously, only small numbers of nucleotides have been recovered from museum and other specimens (e.g. Ash et al., 2017; Kwak et al., 2017). Thus, we sequenced the entire mitochondrial genome of *I. confusus* (five individuals from three localities; refer to Fig. 1, Appendix 1), and four of its relatives: *I. cornuatus*, *I. hirsti*, *I. trichosuri*, and *I. myrmecobii*. We presented the first substantial phylogeny of the subgenera of the genus *Ixodes* (Fig. 7)*.* The subgenera *Sternalixodes* (6 species) and *Ceratixodes* Neumann, 1902 (*I. uriae* White, 1852) were sisters (sister-groups). This is intriguing since *I. uriae* is exclusively a sea-bird tick whereas the eight known species of *Sternalixodes* infest mammals in the adult stage, although birds are apparently the main hosts of the nymphs and larvae in at least two species, *I. cordifer* and *I. hirsti* (Barker and Barker 2022).

It is also intriguing that the subgenus *Exopalpiger* Schulze, 1935 (*I. fecialis* Warburton and Nuttall, 1909) was the sister to subgenera *Sternalixodes* plus *Ceratixodes* [i.e. the arrangement (*Exopalpiger,* (*Sternalixodes, Ceratixodes*))] rather than *Exopalpiger* being closely related to *Endopalpiger* (Fig. 7). Indeed, *Exopalpiger* was well-removed from *Endopalpiger* in our tree (Fig. 7). So, Camicas and Morel (1977) and Camicas et al. (1998) were mistaken when they subsumed *Endopalpiger* into *Exopalpiger*. We can only wonder why Camicas and Morel (1977) and Camicas et al. (1998) made such a major decision without presenting any evidence or argument. The subgenus *Endopalpiger* was the sister-group to subgenera *Sternalixodes* plus *Ceratixodes* plus *Exopalpiger* whereas *Exopalpiger* was the sister to *Sternalixodes* plus *Ceratixodes*. [i.e. ((*Endopalpiger*) (*Sternalixodes, Ceratixodes* and *Exopalpiger*))] (Fig. 7). In other words, *Sternalixodes, Ceratixodes* and *Exopalpiger* shared a Most Recent Common Ancestor to the exclusion of *Endopalpiger*.

The two subgenera in our tree with more than one species, *Endopalpiger* and *Sternalixodes,* were monophyletic (Fig. 7). Regarding *Sternalixodes*, we do not have mt genomes for two of the eight species of this subgenus: *I. cordifer* from Australia and Papua New Guinea*;* and *I. dendrolagi* from Papua New Guinea. We do not expect, however, that mt genomes from these two species will challenge the hypothesis of a monophyletic *Sternalixodes* since *I. confusus, I. cordifer* and *I. dendrolagi* are morphologically similar and thus likely, closely related. Indeed, Wilson (1967) considered *I. confusus, I. cordifer* and *I. dendrolagi* to be so closely related that he designated the *I. cordifer* (species) group for these three species and the associated subspecies of *I. cordifer* (*I. cordifer cordifer* and *I. cordifer bibax*).

Finally, we took the opportunity to make a phylogeny from all 27 of the entire mt genomes that are now available for *Ixodes* (Appendix 2). The subgenera *Sternalixodes, Endopalpiger* and *Ixodes* were monophyletic in our tree (Appendix 2)*.*

### *4.3* Ixodes anatis *Chilton, 1904, the kiwi tick, may be a closely related to the ticks of marsupials of Australia and Papua New Guinea*

The present study of *I. confusus* and its relatives in the subgenus *Sternalixodes* led us to consider *I. anatis*, the kiwi tick, since *I. anatis* was placed in the subgenus *Sternalixodes* by Clifford et al. (1973) but considered best removed from *Sternalixodes* by Kwak and Heath (2018). We made a tree with the *cox 1* fragment (674 bp) from *I. anatis* of Kwak et al. (2017), together with *cox 1* sequences from the mt genomes sequenced by us in the present study, and some *cox1* sequences from GenBank (Appendix 3). Our trees from this short fragment of *cox* 1 had *I. anatis* as a member of the “Australian *Ixodes*” clade. These trees, however, were from only 674 bp of one gene, *cox 1*, of the mt genome. For instance, the unresolved position of *I. uriae* and *I. woyliei* in the *cox 1* tree is resolved by entire mt genomes (Fig. 7). Thus, conclusions about the affinities and evolutionary history of *I. anatis*, the kiwi tick, must await entire mt genome sequences.

## Declaration of competing interest

On behalf of my co-authors I declare that we do not have any conflicts of interest to declare.

## Figures and Tables

**Figure:**
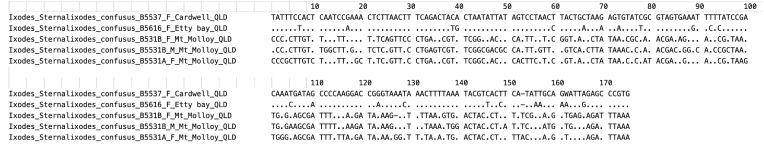


**Figure:**
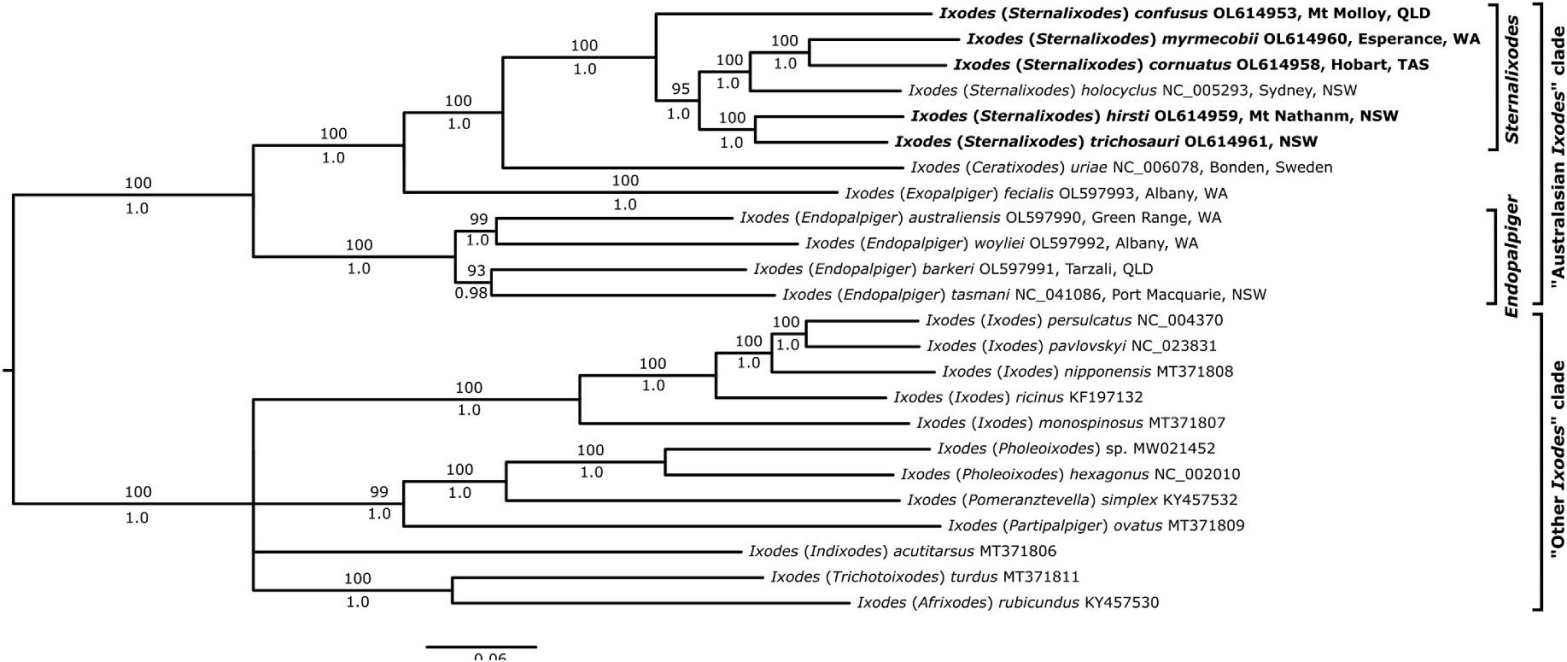


**Figure:**